# Metal nanoparticle-enhanced photocurrent in GaAs photovoltaic structures with microtextured interfaces

**DOI:** 10.1186/s11671-015-0786-6

**Published:** 2015-02-18

**Authors:** Nicolas L Dmitruk, Olga Yu Borkovskaya, Iryna B Mamontova, Sergii V Mamykin, Sergii Z Malynych, Volodymyr R Romanyuk

**Affiliations:** Department of Polaritonic Optoelectronics, V. Lashkaryov Institute of Semiconductor Physics, National Academy of Sciences of Ukraine, 41 Nauky av., Kyiv, 03028 Ukraine

**Keywords:** Metal nanoparticles, Photovoltaic structures, Photocurrent enhancement, Microtextured surfaces, 78.67.Bf, 73.50.Pz, 68.35.Ct

## Abstract

The photocurrent enhancement effect caused by Au and Ag nanoparticles for GaAs-based photovoltaic structures of surface barrier or p-n junction type with microtextured interfaces has been investigated in dependence on the conditions of nanoparticles deposition and, respectively, on the shape and local dielectric environment of obtained nanoparticle arrays. Three nanoparticle deposition methods have been checked: 1) photoinduced chemical deposition of Au from aqueous AuCl_3_ solution forming nanowires on the ridges of quasigrating-type surface microrelief, 2) deposition of Ag nanoparticles from colloidal suspension on the GaAs substrate covered with poly(vinylpyridine), and 3) drop and dry deposition of Au/SiO_2_ core-shell nanoparticles from aqueous colloid solution. The comprehensive investigation of optical reflectance, photoelectric, and electrical characteristics of the fabricated barrier structures has shown the highest photovoltaic parameters for surface microrelief of quasigrating-type and electroless Au nanoparticle deposition. The analysis of characteristics obtained allowed us also to define the mechanisms of the total photocurrent enhancement.

## Background

Deposition of the noble metal (Au, Ag) nanoparticles on the semiconductor surface (interface) is known [1] as a perspective method for increasing the light absorption in semiconductor due to excitation of surface plasmons (SP) in nanoparticles (NP) or surface plasmon polaritons (SPP) in their periodical arrays and nanowires [2]. The spectral range of resonance modes excitation depends on the size and shape of particles, their local environment, and arrangement, which in turn are determined by the method of deposition, the substrate refractive index [3], and surface microrelief [4]. At the same time, texturing the front surface or interface of solar cell (SC) is considered as one of the effective methods to diminish optical losses and to enhance the transmittance of light into semiconductor and the solar cell efficiency [5-8]. In this work, we have investigated the additional photocurrent enhancement effect caused by Au and Ag nanoparticles for GaAs-based photovoltaic structures of surface barrier or p-n junction type with microtextured interfaces [7] in dependence on the conditions of NP deposition and, respectively, on the shape and local dielectric environment of obtained nanoparticle arrays, namely, 1) photoinduced chemical deposition of Au from aqueous AuCl_3_ solution forming nanowires on the ridges of quasigrating-type surface microrelief, 2) deposition of Ag NP from colloidal suspension on the GaAs substrate covered with poly(vinylpyridine), and 3) drop and dry deposition of Au core-shell NP from aqueous colloid solution. The comprehensive investigation of optical reflectance, photoelectric, and electrical characteristics of the fabricated barrier structures has shown the highest photovoltaic parameters for surface microrelief of quasigrating-type and electroless Au NP deposition. The analysis of characteristics obtained allowed us also to define the mechanisms of the total photocurrent enhancement and corresponding increase of solar cell efficiencies.

## Methods

**Figure 1:**
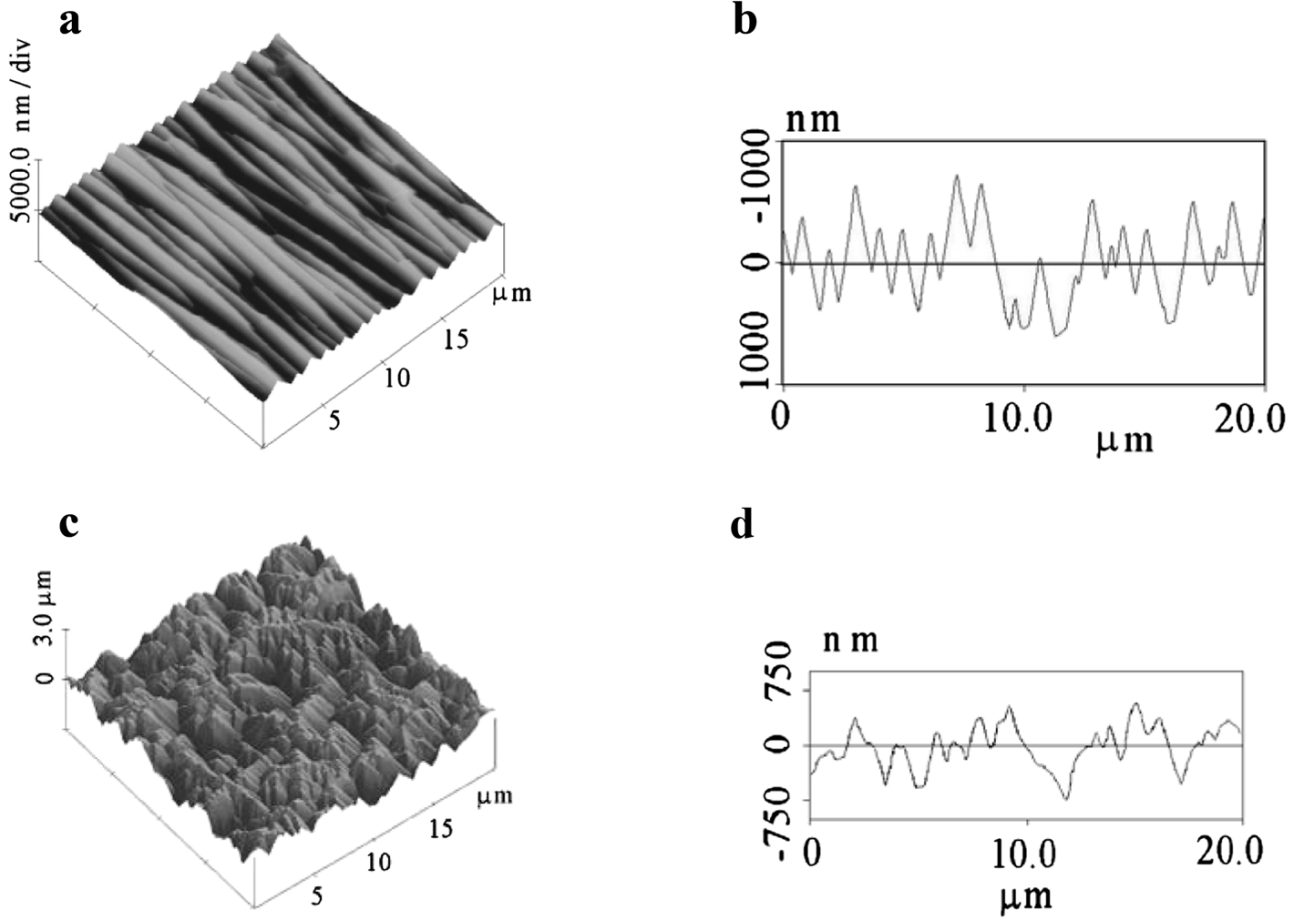
**Surface microreliefs of quasigrating and dendrite-type topologies.** AFM images **(a,** **c)** and section analysis **(b,** **d)** of GaAs surfaces with microrelief of quasigrating **(a,** **b)** and dendrite-like type **(c,** **d)** investigated by AFM technique (Dimension 3000 system with NanoScope IIIA controller, Digital Instruments, Indianapolis, IN, USA) in the tapping mode with a Si3N4 tip.

**Figure 2:**
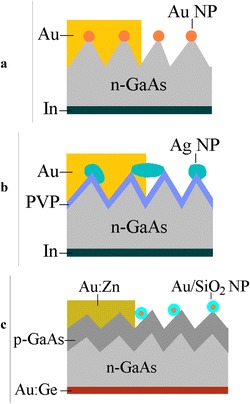
**Schematic diagrams of the photovoltaic structures with textured surface and metal nanoparticles on it.** Au/GaAs structures with Au **(a)** and Ag **(b)** nanoparticles deposited on *n*-GaAs surface by (1) and (2) methods, respectively; p-n-GaAs structure with Au/SiO2 core-shell nanoparticles **(c)**.

Investigated structures have been manufactured on the *n*-GaAs (100) wafers doped to 10^16^ to 10^17^ cm^−3^ with Te. Surface microreliefs of quasigrating and dendrite-type topologies (Figure 1a,b,c,d) were prepared by wet chemical anisotropic etching in 2HF:2H_2_SO_4_:1H_2_O_2_ and concentrated HNO_3_, respectively [7]. Varying the etching conditions (etchant temperature and process duration) allowed us to change both the microrelief depth and mean period of quasigratings. These peculiarities of microrelief are seen in the presented examples of section analysis (Figure 1b,d), and their parameters can be determined by averaging Fourier transformations of AFM data for all cross-sections of AFM image. Three methods of metal nanoparticles deposition were used: 1) photoinduced chemical deposition of Au from aqueous salt AuCl_3_ solution forming nanoparticles of various shape and size located predominantly at the tops of microrelief and, in particular, in the shape of nanowires on the ridges of quasigrating-type surface microrelief [4], 2) deposition of ca. 100 nm Ag NP from colloidal water suspension on poly(vinylpyridine) (PVP) modified the GaAs substrate (for immobilization of NP) with formation of separated NP and aggregates of NP on dielectric PVP interlayer [9], and 3) drop-coating deposition from aqua colloid solution of Au NP of 15 nm core size covered by silica shell with approximately 20-nm thickness [10]. The first two methods were used for the modification of *n*-GaAs surface in Au/GaAs surface-barrier structures when a barrier Au layer formed an ohmic contact with metal nanoparticles. In the last method, the SiO_2_ shell of Au/SiO_2_ core-shell nanoparticles isolated Au nanoparticles from both a semiconductor and metal contact layer. So, it was used in the case of p-n-GaAs structures with metal electrode forming ohmic contact to *p*-GaAs layer. Au/GaAs surface-barrier structures were fabricated by thermal evaporation of semitransparent Au layer through the mask with opening of circular or contact grid form. p-n-GaAs junctions were fabricated by low-temperature (550°C) diffusion of Zn in sealed quartz ampoules with following formation of Au:Zn and Au:Ge ohmic contacts to *p*- and *n*-GaAs layers [8]. The schematic diagrams of structures under the study are shown in Figure 2a,b,c.

## Results and discussion

**Figure 3:**
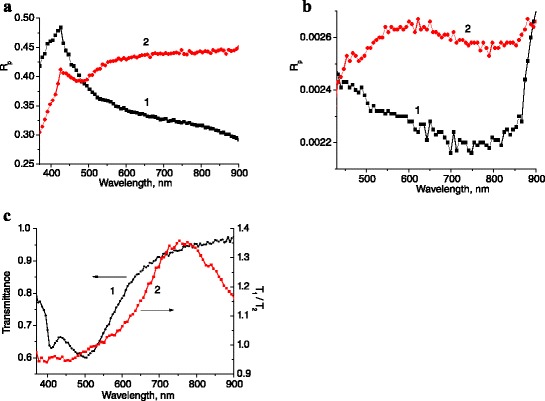
**The spectral dependencies of reflectance (** ***R***_p_ **) and the spectra of light transmittance.** The spectral dependencies of *R*_p_ at near-normal incidence (10°) of light on GaAs with flat surface **(a)** and with surface microrelief of quasigrating type **(b)** before (1) and after (2) Au nanoparticles deposition. **(c)** The spectra of the light transmittance through the layer of aqueous suspension of Ag NP (1) and the ratio of light transmittance through Au/Ag NP/glass and Au/glass structures with Au layer thickness of 30 nm (2).

To determine the change of optical properties of investigated structures due to nanoparticles deposition, the spectra of the light reflection from the flat and microtextured GaAs surfaces before and after nanoparticles deposition were measured (Figure 3a,b). The spectra of specular reflection of p- and s-polarized light were measured at near-normal incidence of light (approximately 10°) on GaAs surface over the range of GaAs fundamental photosensitivity (0.4 to 0.9 μm). In the case of structures with quasigrating type of microrelief, the plane of light incidence was disposed perpendicularly to relief lines. A simplified model of this structure, shown in Figure 2a, where periodic ensemble of metal nanowires with cylindrical shape was situated on the tops of semiconductor surface relief with triangular shape, was used for the simulation of its optical properties (spectra of reflectance, transmittance, and generation rate of electron–hole pairs) [11]. The spectral peculiarities were shown to depend both on the polarization of light, on the depth and period of microrelief, and on the diameter of Au nanowires. Since the microrelief of quasigrating type has some distribution of grating periods, these peculiarities are smoothed. So, the depositions of Au nanoparticles on a flat surface and Au nanowires on the textured one exert both a similar effect on the light reflection spectrum, in particular the increase of *R*_p_ in a long-wave region and minimum at *λ* ≅ 0.5 μm, and a distinct one (Figure 3a,b). The wide maximum at *λ* ≅ 0.6 μm of the curve 2 in Figure 3b suggests the influence of SP/SPP excitation in Au nanowires. Besides, the spectra of the light transmittance through the glass with the same nanolayers of nanoparticles (satellite samples) were also measured (Figure 3c). The spectrum of the light transmittance through the nanolayer with Ag nanoparticles shows properties similar to the ones of transmittance spectrum for the layer of aqueous suspension of Ag NP. So, they are caused by scattering of light by Ag NP due to surface plasmon excitation on them.

**Figure 4:**
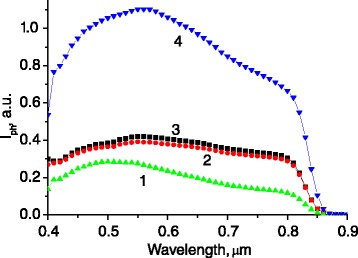
**The spectra of the short-circuit photocurrent for Au/GaAs diode structures.** Structures have flat surface (1) and microtextured one (2 to 4), where (3) - for structures with introduced (PVP/Ag nanoparticles) layers, (4) - for structures with Au nanowires chemically deposited on the ridges of grating-like-type microrelief of GaAs surface.

The optical characteristic peculiarities affect the value and spectral dependencies of the light transmittance into semiconductor and photocurrent of corresponding photovoltaic structures (Figure 4).

**Figure 5:**
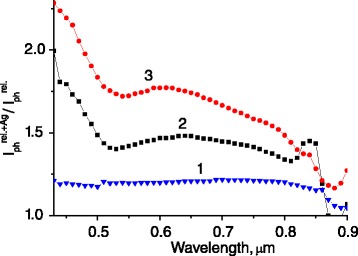
**The spectra of photocurrent enhancement for Au/GaAs diode structures with surface microrelief of quasigrating type.** The structures have Ag/PVP nanolayer (1) and Au nanowire deposition with different mean periods of grating-like microreliefs, 0.76 μm (2) and 1.9 μm (3).

At the same time, a topology of the nanoparticle array (self-assembled 2D array, as in the case of Ag nanoparticles, or 1D nanowires, formed on the ridges of quasigrating microrelief, Au in our case) defines the mechanism of the photocurrent enhancement and its response. Introduction of isolating nanolayers (PVP) changes the electrical characteristics of the structures and considerably diminishes the photocurrent value. So, such method of nanoparticle deposition (Ag) is less effective than electroless method of 1D nanowire formation (Au) (Figures 4 and 5).

In the last case, the photocurrent enhancement is caused not only by surface plasmons excitation in nanoparticles (as in the case of Ag/PVP) but also by surface plasmon polaritons in periodical Au grating. Besides, the Au nanowires, which form barrier contact to GaAs and ohmic contact to Au diode contact, increase the area of photogenerated current carrier collection. That is why the greatest effect is obtained in the case of quasigrating type of surface microrelief and especially when the contact grid lines of Au/GaAs diode are disposed perpendicularly to the Au nanowires on the ridges of microrelief.

**Figure 6:**
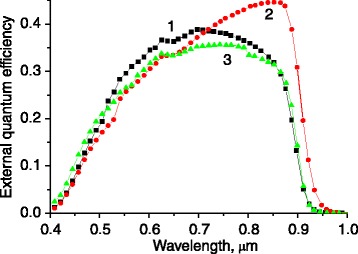
**The external quantum efficiency spectra for the p-n-GaAs structures.** The structures have flat (1), dendrite-like (2) and quasi-grating-like (3) surfaces obtained at a Zn diffusion time of 60 min.

**Figure 7:**
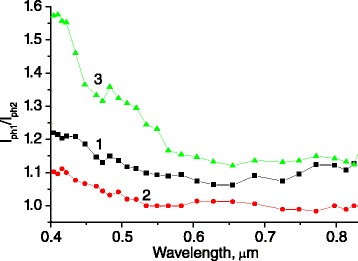
**The spectra of the photocurrent enhancement due to Au/SiO**_2_ **core-shell nanoparticles deposition for GaAs p-n junction structures.** Structures have flat (1) and microtextured surfaces of dendrite-like (2) and quasigrating type (3).

The Au/SiO_2_ core-shell NP deposition also gives rise to photocurrent enhancement, observed for p-n-GaAs photovoltaic structures, which is caused by local plasmon excitation (Figures 6 and 7). It is characteristic that the greatest effect is also obtained for the structures with surface microrelief of quasigrating type.

## Conclusions

Based on the results of the study, the following conclusions can be made:

- It was found that deposition of metal (Au, Ag) nanoparticles on the microtextured surface of GaAs-based photovoltaic structures results in the additional photocurrent enhancement, the value and spectral characteristics of which are dependent both on the microrelief topology and on the method of NP deposition.
- The greatest enhancement effect was obtained for the case of GaAs surface microrelief of quasigrating type, for Au/GaAs surface-barrier form of photovoltaic structures and electroless method of NP deposition resulting in Au nanowire formation on the ridges of microrelief.
- The mechanisms of the Au nanowires caused photocurrent enhancement including both the increase of the light coupling due to SP and SPP excitation and the increase of collection area for photocurrent carriers generated outside the barrier contact.

## Abbreviations

SP: Surface plasmons
NP: Nanoparticles
SPP: Surface plasmon polaritons
SC: Solar cell
PVP: Poly(vinylpyridine)
